# Web-Based Eye Movement Desensitization and Reprocessing for Adults With Suicidal Ideation: Protocol for a Randomized Controlled Trial

**DOI:** 10.2196/30711

**Published:** 2021-11-04

**Authors:** Olga Winkler, Raman Dhaliwal, Andrew Greenshaw, Katie O'Shea, Adam Abba-Aji, Chidi Chima, Scot E Purdon, Lisa Burback

**Affiliations:** 1 Department of Psychiatry Faculty of Medicine and Dentistry University of Alberta Edmonton, AB Canada; 2 Eye Movement Desensitization and Reprocessing International Association Austin, TX United States; 3 Department of Neuropsychology Alberta Hospital Edmonton Edmonton, AB Canada

**Keywords:** suicide, trauma, eye movement desensitization and reprocessing (EMDR), telemedicine, psychotherapy, digital health, eHealth, remote delivery, virtual care

## Abstract

**Background:**

Adversity and traumatic experiences increase the likelihood of suicidal thoughts and behaviors. Eye Movement Desensitization and Reprocessing (EMDR) is an evidence-based, trauma-focused psychotherapy that desensitizes painful memories, so that reminders in the present no longer provoke overwhelming emotional responses. Preliminary evidence suggests that EMDR can be used as an acute intervention in suicidal patients, including those with major depressive disorder. In addition, because of social distancing restrictions during the COVID-19 pandemic, clinicians have been using EMDR on the web and, in the absence of formal evaluations of web-based EMDR, informal reports indicate good results.

**Objective:**

The primary aim of this randomized controlled trial is to investigate whether remotely delivered EMDR (targeting experiences associated with suicidal thinking) reduces suicidal thoughts. Secondary aims include examining the impact of remotely delivered EMDR on symptoms of depression, anxiety, posttraumatic stress, emotional dysregulation, and dissociation. We will also report on adverse events in the EMDR group to explore whether targeting suicidal ideation with EMDR is safe. Finally, we will compare dropout rates between the treatment groups.

**Methods:**

In this randomized controlled trial, 80 adults who express suicidal ideation and meet the study criteria will receive either 12 sessions of twice weekly EMDR plus treatment as usual or treatment as usual alone. EMDR sessions will focus on the most distressing and intrusive memories associated with suicidal ideation. Data for primary and secondary objectives will be collected at baseline, 2 months, and 4 months after enrollment. A subsequent longer-term analysis, beyond the scope of this protocol, will examine differences between the groups with respect to the number of posttreatment emergency room visits, hospitalizations, and overall health care use in the year before and after therapy.

**Results:**

The protocol was approved by the University of Alberta Research Health Ethics Board (protocol ID Pro00090989). Funding for this study was provided by the Mental Health Foundation (grant RES0048906). Recruitment started in May 2021, with a projected completion date of March 2023.

**Conclusions:**

The results of this trial will contribute to knowledge on whether web-based delivery of EMDR is a safe and effective treatment for reducing suicidal ideation and potentially reducing the incidence of suicide attempts in this patient population.

**Trial Registration:**

ClinicalTrials.gov NCT04181047; https://clinicaltrials.gov/ct2/show/NCT04181047

**International Registered Report Identifier (IRRID):**

DERR1-10.2196/30711

## Introduction

### Trauma, Suicide, and Psychopathology

Suicide is the second leading cause of death among those aged 10-29 years and the ninth leading cause of death overall, with reports of 4000 completed suicides per year in Canada [1]. Psychiatric disorders that most strongly predict subsequent suicide attempts are bipolar disorder, posttraumatic stress disorder (PTSD), and major depressive disorder (MDD) [2]. Epidemiological evidence also indicates that adverse or traumatic experiences increase the likelihood of developing both suicidal ideation (SI) and a range of psychiatric disorders [3-9]. Among over 30 psychological risk and protective factors identified for suicidal behavior, the strongest associations were with depression, hopelessness, impulsivity, adverse childhood experiences (ACEs), and trauma [2-9].

The psychological and neurobiological consequences of adverse or traumatic experiences may moderate the development of suicidal thoughts and behaviors [10]. The neurobiological and psychological basis of suicidal thoughts and behaviors is outlined in reviews by van Heeringen and Mann [11] and O’Connor and Nock [12]. ACEs are associated with a strong, graded relationship to later suicide attempts, which may be moderated through stress sensitivity and emotion dysregulation, and expressed as substance use, risky sexual behavior, depressed mood, or anxiety [8,13,14]. Childhood adversity has been associated with deficits in executive function, including attention, working memory, cognitive flexibility, inhibitory control, and emotion regulation. These factors, particularly inhibitory control and emotion dysregulation, are associated with an increased risk for the development of psychopathology and suicidality later in life [15,16]. 

### Eye Movement Desensitization and Reprocessing

Eye Movement Desensitization and Reprocessing (EMDR) is evidence-based therapy, initially developed for PTSD, that desensitizes painful memories, so that present reminders no longer provoke overwhelming emotional responses [17]. EMDR is effective for treating a variety of conditions, including anxiety, depression, trauma, substance misuse, and trauma in patients with severe mental illnesses such as psychosis or bipolar disorder [18-20]. During a typical EMDR session, the client focuses on emotionally disturbing material while bilateral stimulation is applied, either in the form of alternating eye movements, a tactile stimulus such as alternating bilateral tapping, or auditory tones. Standard EMDR uses an 8-phase protocol, including history-taking, preparation, assessment and treatment-planning, desensitization, installation of a positive cognition, body scan, closure, and re-evaluation [21].

EMDR is guided by the Adaptive Information Processing (AIP) model, in which present symptoms are seen as unprocessed explicit and implicit memories stored in the brain that lead to maladaptive information processing and present as posttraumatic and other psychiatric symptoms. In theory, EMDR facilitates the accessing and processing of traumatic memories to an adaptive resolution, after which the disturbing affective distress is relieved, physiological arousal is reduced, negative beliefs are reformulated, and alternative ways of responding to future similar situations are considered [21].

### EMDR and Suicidality

Suicide researchers have hypothesized that suicidal thoughts and behaviors may emerge when environmental triggers activate dimensions of risk in individuals who have been exposed to past adverse experiences and trauma. Two relevant theories include the Escape Theory and the Fluid Vulnerability Theory [22,23]. The Escape Theory suggests that stressful life events activate painful affective states, leading to urges to escape the negative affect and self-awareness. The urge to escape painful affect may lead to reduced self-inhibition, increased passivity, disconnect from emotions, or increased negative thoughts such as suicidal thinking. In this context, SI becomes more accessible and acceptable over time. The more frequent and distressing the suicidal intrusions, the more likely the person is to see them as the best solution to the unescapable, intensely negative state [22].

The Fluid Vulnerability Theory, which focuses on the process of suicide risk rather than risk factors, posits that each person has both a baseline risk state and potential for at least one *suicidal mode*, a time-limited suicidal state with individual characteristic features related to the person’s suicidal belief (cognitive) system, affective system, physiological system, and behavioral (motivational) system, which together work in synchrony. This theory proposes that the risk state and suicidal mode can be activated by either external or internal triggers, and usually ends in a state “characterized by specific or core cognitive themes (i.e., unlovability, helplessness, poor distress tolerance, and perceived burdensomeness), acute dysphoria and related physiological arousal (ie, Axis I symptomatology), and associated death-related behaviors” [23]. During these *suicidal modes*, motivational and behavioral systems may be engaged which activate specific motoric and physiologic responses, for example, fight, flight, or freeze, along with preparatory urges or behaviors. Sometimes, these states are misinterpreted cognitively as a threat in themselves, leading to escalation of distress. These modes are based on the original cognitive therapy model by Beck [24] and defined as “specific suborganizations within the personality organization (that) incorporates the relevant components of the basic systems of personality: cognitive (or information processing), affective, behavioral, and motivational.” Beck [24] described a mode as an “integrated cognitive-affective-behavioral network [that] produces a synchronous response to external demands and provides a mechanism for implementing internal dictates and goals” [23]. The Fluid Vulnerability Theory also assumes that a person’s baseline level of risk is determined by historical and developmental factors that predict why activation of a suicidal state might occur in a particular context and with a particular intensity. This vulnerability also has cognitive, affective, physiological, and behavioral aspects, and improvements in any one area can reduce vulnerability across the system. This theory emphasizes the cognitive *suicidal belief system*, which may stem from historical factors such as adversity. Rudd [23] proposed that the suicidal belief system is “potentially amenable to change during periods of activation, [and] activation is critical to treatment progress and success.”

Both the Escape Theory and the Fluid Vulnerability Theory are compatible with the AIP model and the Working Memory Model of EMDR and may provide theoretical support for why EMDR could reduce suicidality [21,25]. In the AIP model, stressful and especially overwhelming experiences are conceptualized as affectively laden memories with explicit and implicit components that are incompletely processed. Multiple stressful experiences may be associated within networks linked by common themes, cognitions, emotions, implicit states, urges, or other similarities. These memories include a cognitive component, which may be the consequence of dysfunctional learning or overgeneralization, that may contribute to core beliefs. Activation of these memory networks, along with their cognitive, emotional, sensory, physiological, and behavioral components, represent the basis for symptomatology [21]. The Working Memory Theory posits that memories are transferred to working memory during EMDR and that eye movements function to reduce the vividness and intensity of memory-related imagery, partly because they *tax* working memory by using processing resources in the visuospatial sketchpad, which reduces the emotionality of the memory. This is corroborated by research showing reduced amygdala activation during EMDR [26]. At the same time, while the experience is held in awareness in working memory, it is amenable to change and is ultimately reconsolidated into a different form, with altered and more adaptive meta-cognitive interpretations [25].

Standard EMDR begins with accessing past associations to a current presenting problem by asking the participant about their current cognitions, emotions, or sensations. Holding all these elements together in awareness, the therapist then directs the person to *float back* to earlier times in life when these elements were experienced together, thus finding implicit past associations with the presenting problem. Alternatively, patients are asked to provide experiences that have *proven* their core beliefs, for example, *I am unlovable*, which are likewise processed. The targeted memory is desensitized and then paired with a positive core belief, which allows the person to access more adaptive information. Once the past experience is processed, present reminders and future fears of when a similar experience may happen are found in a similar fashion and then again processed. The Escape Theory would suggest that if EMDR desensitizes distressing memories associated with painful affective states fueling suicidal intrusions, SI would decrease. From the Fluid Vulnerability Model perspective, EMDR may address multiple aspects of the *suicidal mode*, as well as baseline risk, by targeting all 4 components (cognitive, affective, physiological, and behavioral) of the experiences contributing to risk. As the suicidal belief system shifts and internal and external cues no longer provoke arousal, vulnerability to activating future suicidal states may decrease. As cognitive, affective, and sensory (somatic or physiologic) aspects are addressed, in past, present, and future time frames, we hypothesize that EMDR will be able to uniquely access and address multiple dimensions of risk for suicidality concurrently in an individualized manner.

Preliminary evidence indicates that EMDR may be an effective intervention for SI [27-29]. With populations experiencing suicidal thoughts, EMDR may decrease SI, even when SI is not addressed directly, as is the case when PTSD, anxiety, or depression are primary treatment targets [27,28,30,31]. Most recently, Fereidouni reported a reduction in the Beck Scale for Suicide Ideation (BSS) scores in a randomized controlled trial (RCT) using intensive EMDR in 70 adult inpatients with MDD. Participants in the intervention group received individual EMDR for 45 to 90 minutes 3 times per week for 9 sessions. In that study, the mean BSS score dropped significantly from 26.48 to 11.11 in the EMDR group compared with no change in the control group. However, no other outcome measures were reported, and the participants were limited to those with depression [28]. Currently, most psychotherapeutic treatments target a specific diagnosis. Examples include EMDR treatment for PTSD and Dialectical Behavioral Therapy for borderline personality disorder (BPD), and changes in SI are usually reported as secondary outcomes [17,32]. Given the increasing public health need to improve treatment options for suicidality, this pragmatic real-world study was designed to assess the impact and safety of using EMDR to target SI across a wide spectrum of diagnoses.

### Web-Based Delivery of EMDR

The COVID-19 pandemic has forced a rapid shift from in-person psychotherapy to remotely delivered psychotherapy services, both to reduce the spread of COVID-19 and to maintain service accessibility. This rapid shift to web-based care has raised concerns about whether therapy delivered via the web is as safe and effective as in-person therapy. A recent systematic review reported level 1a evidence that remote access, digitally delivered trauma therapies such as prolonged exposure therapy, cognitive processing therapy, and therapeutic exposure can be as effective as in-person treatment and may improve access to care [33]. Although there is a paucity of research on the safety and effectiveness of web-delivered EMDR, remotely delivered EMDR has been adopted clinically around the world [34]. This project will contribute to this area by exploring, with a focus on SI, whether remotely delivered EMDR can be delivered safely and effectively in a routine clinical practice setting. Remote access, rather than a face-to-face approach, has been chosen for our study as, locally in Edmonton, Alberta, Canada, the COVID-19 pandemic resulted in an increase in demand for mental health services at the same time as a reduction in the availability of in-person mental health support. Furthermore, public health measures have necessitated periodic self-isolation, leading to clinic cancelations. For these reasons, this project will deliver EMDR via end-to-end encrypted Zoom videoconferencing (Zoom Video Communications, Inc), rather than in person.

### Objectives

This study aims to examine whether web-based delivery of EMDR reduces the intensity of SI in adults, as measured by the BSS and the Columbia Suicide Severity Rating Scale (CSSRS). We hypothesize that targeting memories that are associated with SI, including addressing the associated suicidal belief system, will reduce distress and emotional dysregulation driving SI.

SI is often associated with mood, anxiety, and posttraumatic symptoms, and emotion dysregulation and dissociation are common in populations experiencing intense negative emotional states and those at risk of SI, such as PTSD and BPD [35-37]. The secondary study objectives, therefore, include measuring the impact of our modified EMDR treatment on symptoms of depression, anxiety, posttraumatic stress, emotional dysregulation, and dissociation. Measuring these symptoms will allow better characterization of our study sample and allow comparison with previous literature. In addition, we wish to learn if focusing on SI-associated experiences specifically leads to improvement in these common comorbid symptoms. Previous literature indicates that reductions in SI that occur during PTSD treatment, for example, may be mediated by improvements in PTSD or depressive symptoms [38]. It is unknown whether focusing specifically on SI, rather than a specific diagnosis such as PTSD, would also result in decreases in mood, anxiety, and PTSD symptoms.

A further objective is to report on the history of ACEs and the level of dissociative symptoms experienced by study participants (EMDR vs treatment as usual [TAU] group), as these may be markers of complexity [39,40]. Dissociative symptoms have been linked to increased comorbidity, exposure to childhood adversities, clinical severity, and lower response to trauma-focused therapies (TFTs) [39-42]. There is controversy as to whether dissociation is a barrier to using TFTs, such as EMDR [43]. The dissociative subtype of PTSD has been associated with midline prefrontal inhibition of limbic regions involved in emotion regulation, leading to emotional overmodulation [42]. One possible clinical implication is that such patients may have difficulty improving with TFTs because of impaired emotional regulation capacities and a tendency to dissociate upon exposure to distressing cues inherent in the processing of the trauma. This could impair the ability to adequately activate the fear network, leading to reduced effectiveness of TFTs [42]. Therefore, measuring dissociation at baseline and after treatment will provide important information about whether EMDR impacts dissociation, or if dissociative symptoms adversely impact treatment response.

We will compare dropout rates between treatment groups and report on any adverse events that arise in the EMDR group to explore the safety of using EMDR to target SI. Experts in the field of trauma have long believed that survivors of trauma should be treated using a phased approach [44-46]. The first phase focuses on stabilization and the introduction of coping skills to reduce self-harm and suicidality. Once phase 1 is completed and the person is no longer a risk to themselves or others, phase 2 may begin, with TFTs such as EMDR, which focus on distressing memories directly. However, this phased approach emphasizing stabilization before trauma processing has been criticized as lacking evidence [47]. Therefore, studies reporting on the safety of TFTs, such as EMDR, in patients with SI can help address this controversy.

## Methods

### Study Design

**Figure 1:**
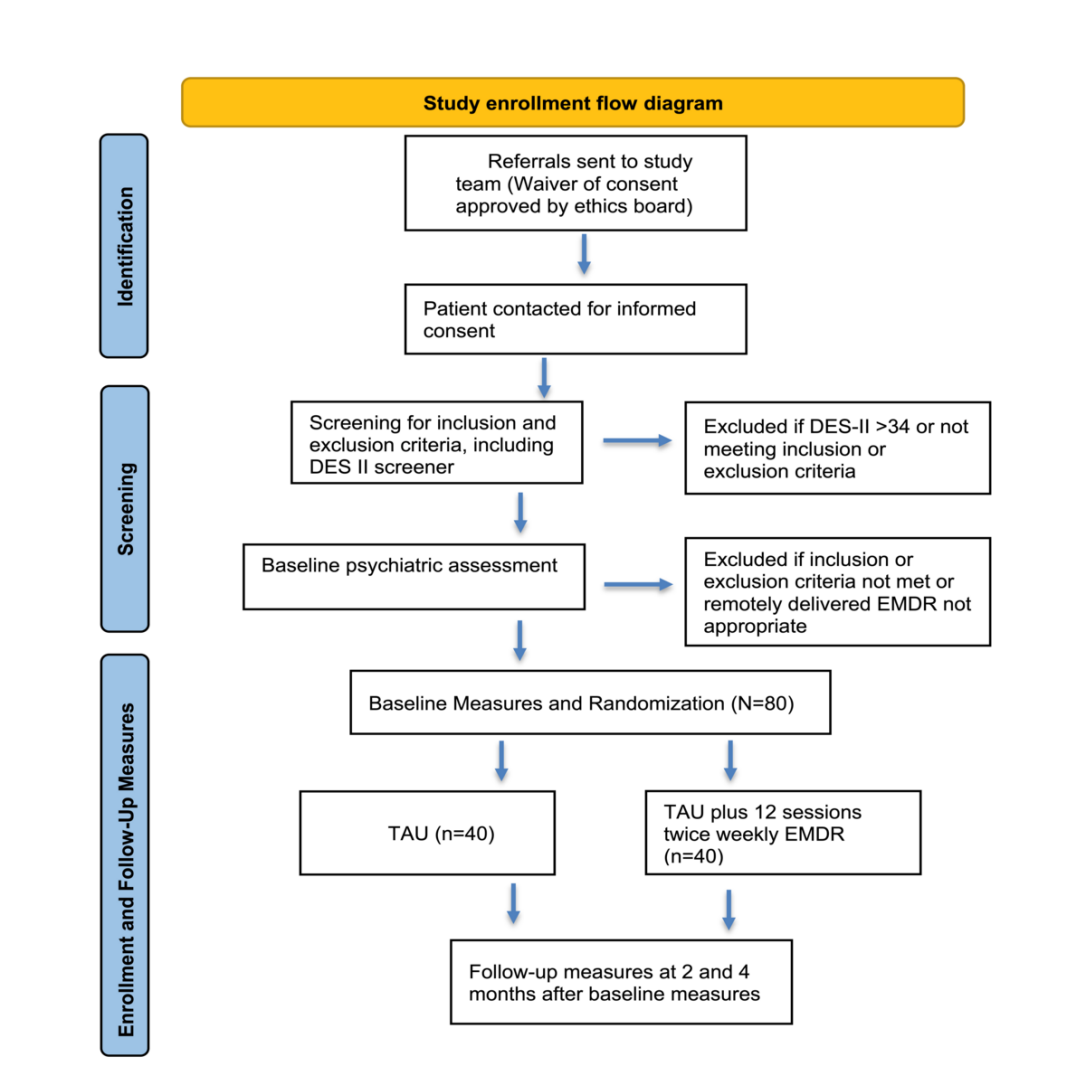
Flow diagram. DES-II: Dissociative Experiences Scale-II; EMDR: Eye Movement Desensitization and Reprocessing; TAU: treatment as usual.

The study is a nonblinded RCT that will evaluate the effects of remotely delivered EMDR in combination with TAU, compared with TAU alone for adult patients with SI. Owing to the nature of EMDR, trial blinding to the research team and clinical staff is not possible. All clinical contact will occur on the web via a health care–level encrypted Zoom platform. The anticipated flow of participant enrollment is shown in Figure 1, and details are included in the *Study Procedure* section. Participants will be randomized (by computer-generated random allocation) to receive either intensive 90-minute EMDR sessions twice per week plus TAU or TAU alone. A pilot study by Proudlock et al [29] and an RCT by Fereidouni et al [28] used a similar design, with intensive EMDR provided 2 to 3 times per week. In the study by Proudlock et al [29], most of the participants were outpatients with an acute mental health crisis with SI. In the RCT, the participants were inpatients with major depression and suicidal thoughts [28]. The literature suggests that intensive (ie, multiple sessions per week) therapy is safe and effective and may reduce attrition [28,29,48,49].

The primary outcome is the intensity of SI in adults, as measured by the BSS and the CSSRS. Secondary outcomes include mood (Beck Depression Inventory-II and Patient Health Questionnaire 9), anxiety (Generalized Anxiety Scale-7), posttraumatic symptoms (Impact of Events Scale Revised), dissociation symptoms (Dissociative Experiences Scale-II [DES-II]), and emotion dysregulation (Difficulties in Emotional Regulation Scale). Secondary outcomes include adverse events and dropout rates. A subsequent longer-term analysis, beyond the scope of this protocol, will examine differences between groups with respect to the number of emergency room visits, hospitalizations, and overall health care use in the year before and after therapy.

### Ethics

The Health Research Ethics Board at the University of Alberta approved the study protocol (protocol ID number: Pro00090989). The study is registered at ClinicalTrials.gov (ID number: NCT04181047). Although EMDR is a gold standard, evidence-based treatment for trauma [17,18], current practice guidelines do not generally endorse EMDR specifically for the treatment of suicidal thinking, and data on remote delivery of EMDR are limited [33]. Some providers believe that trauma therapy should not be attempted in patients with SI, based on fears that exposure to traumatic memories may increase emotional dysregulation or worsen suicidality. EMDR may, in some cases, lead to temporarily increased PTSD symptoms, anxiety, nightmares, or distress during treatment. The web-based nature of the treatment may also add privacy and safety risks because of the use of electronic communications and the fact that the therapist is not in the same location as the participant. Therefore, clinical safety procedures were developed to monitor and manage increased SI and adverse events, in addition to ensuring informed consent from participants. These considerations were discussed with the Health Research Ethics Review Board, which approved the study.

### Participants

Adults (aged 18-65 years) with SI in the last week are eligible for this study. SI may be chronic or acute and of any intensity as long as it is not accompanied by an active plan with intent, given the concerns about immediate safety and the need for stabilization in this population. As SI can occur across various diagnoses, participants are not limited to having one main diagnosis. Participants with suicidal thoughts and any or all of the following primary diagnoses: mood and anxiety disorders, trauma and stress-related disorders, or personality disorders as primary diagnoses are eligible for this study. Participants must also be willing and able to volunteer to participate in the study, provide informed consent, and follow up twice weekly for EMDR sessions if they are randomized to the EMDR group (a total of 12 desensitization sessions). Participants must have a primary service provider, either a physician or a mental health professional, who they can access for care outside of EMDR sessions. Participants must have access to their own laptop or desktop computer that enables bilateral stimulation with a working screen, camera, and microphone, as well as access to a quiet, private, well-lit space for therapy. Participants must be willing to refrain from benzodiazepine, cannabis, or illicit substance use in the 24 hours before or after EMDR sessions to avoid interference with EMDR and memory consolidation. Participants must also be willing to adhere to the study safety precautions (see the *Clinical Safety Procedures* section).

Participants will be excluded from the study if, at the time of the baseline assessment, SI is accompanied by intent or a plan to follow through with suicide. The rationale is that those with intent or a plan may be more at risk of imminently following through with acting on the ideation. Clinical guidelines recommend that this warrants inpatient stabilization to ensure immediate safety [50]. Participants will be excluded if they score above 34 on the DES-II or report severe dissociative symptoms during the baseline psychiatric assessment interview in keeping with a separate dissociative disorder, such as hearing internal voices, amnestic episodes, dissociative fugue states, passivity experiences, first rank symptoms under stress, the subjective experience of having alter personality self-states, or severe isolation of affect, with the inability to feel body sensations or emotions. Clinical experience and the scientific literature suggest that severe dissociative symptoms signal a poor response to standard EMDR therapy or require special techniques or extensive stabilization. Participants with manic or psychotic symptoms will be excluded to reduce heterogeneity, as are those undergoing electroconvulsive therapy, which may have an impact on memory. Participants undergoing or planning to undergo another trauma-focused psychotherapy in the 4-month study period will also be excluded to reduce bias. Participants who are known to be pregnant will be excluded, as there is limited information about the impact of EMDR in pregnancy.

### Study Sample Size and Duration

The only analogous trial published, to our knowledge, is an RCT by Fereidouni et al [28], which also used the BSS to measure changes in SI, but in a depressed inpatient population. They reported a required sample size of 31 per arm, which was increased to 35 to account for attrition, calculated using a CI of 95%, a statistical power of 80%, and a minimum clinically significant difference of 5%. This previous trial enrolled 70 participants and had no dropouts.

In this study, the overall target sample size is 80 participants (40 in each group). To detect a within-group change (pre- and posttreatment changes in rating scale measures) of Cohen *d*=0.50, applying a 2-tailed α level of .05 and power at 0.80 (0.75 for between-group changes), the study will require 32 participants in each group. Our target sample size of 40 participants per group was chosen to account for an anticipated attrition rate of 20%, resulting in samples no smaller than 32. The attrition rate is based on clinical experience, as well as the literature reporting a mean dropout rate of approximately 18% in previous EMDR trials [29,51].

### Clinical Safety Procedures

To ensure participant safety during the study, the following measures have been instituted:

1. All participants must have access to a health professional, such as a family physician or psychiatrist, during the course of this trial, who is willing to provide general mental health care, as necessary. If a safety concern arises during the study, the participant’s provider will be informed.
2. Before commencing EMDR, the participant will confirm their address, phone number, email address, and emergency contact person. This is necessary so that an emergency response can be activated if clinically indicated.
3. If the participant is enrolled in the EMDR treatment group, the study therapist will ensure that a written safety plan has been completed before treatment is initiated. This safety plan will include helping the participant to identify their warning signs for crisis, their internal coping resources, sources of helpful distraction, and helpful others, including professional resources, from whom they can access assistance in a crisis. Contact information for Edmonton crisis services will also be provided.
4. To ensure safety during EMDR sessions, participants will be asked to ensure that there is a supportive person who will be available to assist within 5 minutes, in the unlikely case of an emergency.
5. If there is a significant worsening in SI, the study therapist may pause EMDR and focus on crisis stabilization and the institution of the individualized safety plan.
6. The study research assistant (RA) was trained in a protocol for enrolling participants, which includes a safety protocol to manage any unanticipated situations in which a participant spontaneously expresses worsening or active SI (Multimedia Appendix 1). Consent for EMDR therapy includes an understanding that in the case of imminent risk of harm to self or others, the therapist or RA may need to activate the safety plan or call emergency services.
7. For the EMDR group, adverse events will be queried and recorded at the beginning of each EMDR session, along with recording a self-report of the intensity of various symptoms, including SI, on a 0-10 scale. As therapy is taking place in the context of clinical care, progress will be documented in the electronic health record, which may be accessed by the participants’ treatment team (see Multimedia Appendix 2 for the items captured regarding adverse events). In addition, serious adverse events will be directly reported to the participants’ treatment team. Each person participating in the study must agree to maintain a relationship with his or her community treatment team during the study to avoid a situation where the person has no access to care during or immediately after participating in the study.

Participant confidentiality and web-based security:

1. Informed consent for web-based EMDR therapy also includes an agreement that the patient will disclose their physical location, keep a working telephone with them in the case of internet disconnection, and connect in a private area. Health service–level encrypted Zoom, as authorized by Alberta Health Services, will be used for videoconferencing therapy to minimize security concerns. The participants will also be encouraged to use their own private computer and Wi-Fi.
2. Encryption will be used in the case that email is needed to send potentially identifying information, in accordance with Alberta Health Services policy.

### Study Procedure

This study is being conducted in partnership with the outpatient mental health clinics of the Alberta Health Services Addiction and Mental Health, Edmonton Zone. Figure 1 shows the anticipated flow of subject enrollment and assessments.

1. Community clinicians will make a referral through email, fax, or phone to the RA. The research ethics board at the University of Alberta approved a waiver of consent to allow the RA to receive and screen referrals and contact the potential participants to set up a Zoom meeting to explain the study and obtain informed consent. If necessary, the RA can aid the potential participant in setting up Zoom and instructing the person on its use. During this initial Zoom meeting, the RA will obtain informed consent for participation in the study, which will be collected and managed using REDCap (Research Electronic Data Capture) tools hosted by the Women & Children's Health Research Institute at the University of Alberta, using a 2-factor authorization process [52]. Participants will be informed that they may withdraw their consent and opt out of the study at any time during the 4-month study period, whereas participant data may be withdrawn at any point before treatment begins.
2. After obtaining consent, the RA will send a link from REDCap to the participant to complete the DES-II electronically. A DES-II score ≥34 will exclude a person from the study. If enrollment screening criteria are met, the participants will receive a psychiatric assessment by the study psychiatrist. The purpose of the psychiatrist assessment is to complete an in-depth assessment to rule out contraindications to web-based EMDR and to ensure eligibility criteria are met, including ruling out severe dissociation not apparent on the DES-II screening questionnaire. The psychiatrist will also perform a baseline diagnostic assessment according to *Diagnostic and Statistical Manual of Mental Disorders - 5* criteria to evaluate the baseline diagnoses. If the person appears suitable for the study, baseline self-report measures will be completed electronically (Table 1). Once the baseline measures are complete, REDCap will randomly assign the person to either the EMDR group or the TAU group using random computerized allocation.
3. All patients will complete baseline and follow-up measures electronically through REDCap, as shown in Table 1.
4. Patients in the EMDR treatment group will receive live, twice weekly EMDR through Zoom videoconferencing. The study therapist will take a relevant history, provide limited psychoeducation, and explain EMDR to the patient. After developing a safety plan with the participant, up to 5 standard preparation exercises will be completed before therapy (container, safe state, internal meeting place, safe place for parts, and updating the emotional circuits [53]). Patients will then receive EMDR, targeting the experiences or core beliefs associated with the SI. The standard EMDR protocol will generally be used, with the modification that the *future template* will be a *flashforward* of the *worst-case scenario future when suicide would again seem like an option*. Although the standard *future template* involves running a mental movie about the future, a *flashforward* targets the person’s mental representation of future events in a similar fashion as past events are targeted (see the paper by Logie and De Jongh [54] for details about this strategy).

**Table 1:** Timing and content of study measures.

Measure	Content of measure	Timing of measures		
		Baseline	2 months after enrollment	4 months after enrollment
Demographic questionnaire	Demographics	✓^a^		
ACES^b^	Adverse childhood experiences	✓		
DES-II^c^	Dissociative symptoms	✓		✓
BSS^d^	Suicidal ideation	✓	✓	✓
CSSRS^e^—clinician rated	Suicidal ideation	✓		
CSSRS—past week (self-rated)	Suicidal ideation	✓	✓	✓
BDI-II^f^	Depressive symptoms	✓	✓	✓
PHQ-9^g^	Depressive symptoms	✓	✓	✓
GAD-7^h^	Anxiety	✓	✓	✓
IES-R^i^	PTSD symptoms	✓	✓	✓
DERS^j^	Emotional dysregulation	✓		✓

^a^Indicates the timing of the respective measures.

^b^ACES: Adverse Childhood Experiences Scale.

^c^DES-II: Dissociative Experiences Scale.

^d^BSS: Beck Scale for Suicide Ideation.

^e^CSSRS: Columbia Suicide Severity Rating Scale.

^f^BDI-II: Beck Depression Inventory-II.

^g^PHQ-9: Patient Health Questionnaire-9.

^h^GAD-7: Generalized Anxiety Disorder-7.

^i^IES-R: Impact of Events Scale Revised.

^j^DERS: Difficulties in Emotional Regulation Scale.

Some other modifications to the standard protocol are allowed. Specifically, intrusive or distressing memories may be targeted initially, if needed, instead of the first, worst, current, and future order of the standard protocol. If there is apprehension about doing EMDR, a flashforward of the worst thing that could happen may be used to address this resistance before targeting memories. This was reported as a successful strategy in an intensive treatment program [49]. In addition, therapists can use the EMDR early trauma protocol if there are significant attachment problems, add additional dual attention tasks to load working memory, or use shorter sets of bilateral stimulation if the standard protocol is not tolerated [55,56]. Strategies in the Jim Knipe EMDR Toolbox can also be used as needed [57]. Modifications to the standard protocol will be recorded in REDCap and reported on.

EMDR will specifically target the traumatic memories or core beliefs associated with the SI. These targets may be easily identified by the patient in some cases. Alternatively, the standard floatback method may be used to identify memory targets, or therapists may target the somatic urge or state associated with suicidal thoughts. This strategy of targeting states or urges has been utilized in EMDR protocols such as the *DeprEnd protocol* for depression and the *DeTUR protocol* for urges associated with substance use disorders [58,59]. Other possible targets for EMDR include memory of the circumstances surrounding the first occurrence of SI, memories at the origin of the negative beliefs associated with SI, or memories related to hopelessness and despair [21,58,60]. If escape fantasies, including the fantasy of escaping through suicide, emerge during memory processing, the participant may be encouraged to notice the fantasy rather than avoid or suppress it. In addition, if nightmares arise during the course of treatment, they can also be targeted directly using EMDR if clearly related to SI or the experiences being reviewed in therapy sessions.

Participants will be seen twice weekly until the therapy is completed (12 desensitization sessions in total). Symptoms and any adverse reactions will be recorded at the beginning of each session using a standard EMDR session progress note form.

### Measures

#### Primary Outcome Measures

##### Beck Scale for Suicide Ideation

The BSS is a 21-item questionnaire on SI and behavior over the past week. The score ranges from 0 to 42, with higher scores indicating worse outcomes. Questions 6 through 19 are not completed if answers to both questions 4 and 5 indicate that the person has no suicidal desire and would try to save their life if in a life-threatening situation. Question 20 asks about previous suicide attempts, and question 21 asks about the wish to die during any such attempt [61] (Digital adaptation 2021 NCS Pearson Inc. All rights reserved. Adapted and used under license #LSR-262494).

##### Columbia Suicide Severity Rating Scale

The CSSRS is a questionnaire used for suicide assessment, developed by multiple institutions including Columbia University. Several versions exist; this study will use the clinician-rated version that assesses lifetime and recent SI (last week) and suicidal behavior at baseline. In addition, a self-report version will be used at baseline, 2 months, and 4 months to assess SI in the *past 1 week*, which includes 5 questions about SI and 2 questions about suicidal behavior [62].

#### Secondary Outcome Measures

##### Adverse Childhood Experiences Scale

The Adverse Childhood Experiences Scale is a standard 10-item questionnaire that assesses the presence or absence of adversities experienced in the first 18 years of life, including emotional, physical, or sexual abuse, neglect, parental divorce, domestic abuse, familial substance abuse, incarceration, or mental illness. Higher scores are indicative of more childhood adversity and have been consistently associated with an increased risk of psychiatric illness, substance abuse, and physical illness [63].

##### Dissociative Experiences Scale-II

The DES-II is a 28-item questionnaire that includes questions about common dissociative symptoms, which are scored based on the frequency of experiencing the symptom, from 0% of the time to 100% of the time. A higher score (range 0-100) indicates more severe dissociative pathology. The mean scores for PTSD, Dissociative Disorder Not Otherwise Specified, and Dissociative Identity Disorder in a previous study were 31, 36, and 48, respectively [64].

##### Beck Depression Inventory-II

The Beck Depression Inventory-II is a 21-item questionnaire focusing on symptoms of MDD, including one question on SI or wishes. Each question is scored on a 0-3 scale, with higher scores indicating a higher likelihood of MDD [65] (digital adaptation 2021 NCS Pearson Inc. All rights reserved. Adapted and used under license #LSR-262494).

##### Patient Health Questionnaire-9

The Patient Health Questionnaire-9 Self-Report is a self-report questionnaire for assessing depressive symptoms during the previous 2 weeks, using a 4-point Likert scale to indicate symptom frequency for each item (0=not at all; 3=nearly every day). Higher scores (range 0-27) indicate more severe depressive symptoms. Included is also a question about how difficult the symptoms made it for the participant to work, take care of things at home, or get along with people (rated from not difficult to extremely difficult, on a 4-point scale) [66].

##### Generalized Anxiety Disorder 7

The Generalized Anxiety Scale-7 is a 7-item self-report scale for anxiety symptoms, with each symptom rated for the past 2 weeks on a 4-point scale. The scale ranges from 0 (not at all) to 3 (nearly every day), with higher scores indicating worse anxiety symptoms. There is also a question about how difficult the problems endorsed made it for the participant to work, take care of things at home, or get along with people (rated from not difficult to extremely difficult, on a 4-point scale) [67].

##### Impact of Events Revised

The Impact of Events Scale Revised is a 22-item questionnaire, which rates the intensity of distress over the past 7 days, related to a past event. Symptoms related to distress are rated on a 5-point scale, from 0 for *not at all* to 4 for *extremely* distressing, with scores ranging from 0 to 88. Questions include symptoms generally indicative of posttraumatic stress, with a higher score indicating more severe symptoms [68].

##### Difficulties in Emotional Regulation Scale

The Difficulties in Emotional Regulation Scale is a 35-item questionnaire focusing on symptoms related to emotion regulation. Questions are rated on a 5-point scale, and participants rate how often the statements apply to them by providing a number, where 1 indicates *almost never* (0%-10% of the time) and 5 indicates *almost always* (91%-100% of the time) [69].

##### Session Forms

For the EMDR group, session forms will be used to track weekly progress. These forms include a scale of 0 to 10 (ranging from no difficulties to highest intensity) to record the intensity of suicidal thoughts, self-harm urges, and the following symptoms: worry thoughts, anxiety, guilt or shame, anger, sadness, flashbacks, sleep problems, substance use, suicidal thoughts or impulses, self-harm urges, concentration difficulties, lethargy or fatigue, appetite problems, and repetitive thoughts. These forms will also capture the session focus and any deviations from the standard EMDR protocol.

### Statistical Analysis

Treatment groups will be compared for differences in demographics or clinical severity that may be relevant to differences in outcomes. The number of desensitization sessions will be reported, along with any adverse events.

The main statistical contrast will compare measures for the EMDR versus the TAU group; particular emphasis will be placed on the primary outcome variables of severity of SI before and after treatment in each group. Pre- and posttreatment effects on rating scales will be analyzed using parametric (2-tailed *t* tests and analysis of variance [ANOVA]) or nonparametric (Mann-Whitney and Wilcoxon and Friedman or Kruskal-Wallis ANOVA) tests, where appropriate. For multiple comparisons in the analysis, the error rates will be adjusted appropriately using Bonferroni corrections. ANOVA followed by multiple comparison tests will be applied where the number of within-subject test times k>2 (Table 1). For single time measures, for example, Adverse Childhood Experiences Scale or clinician-rated suicide ratings, simple contrasts will be assessed with *t* tests or nonparametric equivalents, as appropriate. The statistical criterion for type one errors will be a 2-tailed probability of *P*≥.05, after appropriate adjustment for multiple comparisons. 

## Results

It is anticipated that the active recruitment, psychotherapy treatment, and data collection phase of this study will take 18 months to complete. We expect to report the primary and secondary outcomes by mid-2023. The primary outcome will be changes in SI; secondary outcomes will include changes in reported depressive, anxious, dissociative, and PTSD symptoms, as well as changes in emotional dysregulation. In addition, we will report on dropout rates and adverse effects that emerge during EMDR treatment. Study participants will be informed about the trial results via a plain language summary that will be sent to them. Academic papers and summary reports will be provided to the Mental Health Foundation for knowledge dissemination. Evidence regarding the safety and efficacy of EMDR in the context of SI will be discussed with Alberta Health Services and presented in clinical academic settings to support knowledge translation and knowledge implementation.

## Discussion

### Principal Hypotheses

There exists a significant body of literature demonstrating that childhood and adult adverse experiences are strongly associated with SI, suicide attempts, self-injurious behavior, and the development of a wide range of psychiatric illnesses [3-9]. If the AIP model of EMDR is correct, experiences lead to the development of explicit and implicit memories that drive or contribute to painful core beliefs or overwhelming affect. We hypothesize that targeting these memories directly will provide a direct treatment for emotional dysregulation and suicidal thinking.

Current treatment of SI usually focuses on treating comorbidities such as depression and teaching new ways of coping, thinking, or behaving. None of the currently recommended treatments for suicidality target memories directly. EMDR desensitizes the emotionality of traumatic memories, followed by reprocessing the associated negative core belief with a more adaptive one. A recent RCT using EMDR for suicidal thoughts in inpatients with depression offers the first RCT evidence that EMDR can specifically reduce suicidal thoughts [28]. This adds to the uncontrolled data that suggest that EMDR can reduce suicidality in patients in crisis or those with suicidal thinking [27,29]. This study aims to target SI from a transdiagnostic perspective, focusing on the memories driving or associated with the SI across a broad spectrum of diagnoses.

### Implications for the Future

If the study results support the use of EMDR as a safe and effective treatment for people with SI, it would challenge current clinical norms. The PTSD literature suggests that treating PTSD with TFTs reduces SI, even after controlling for depression and hopelessness [70,71]. However, clinicians are often reluctant to offer TFT in suicidal patients for fear of worsening their suicide risk. Therefore, patients’ trauma symptoms may go untreated or be addressed solely with medications, and they may experience repeated bouts of crisis or hospitalization, leading to further demoralization. This study may provide evidence to support clinicians in using TFTs for patients with SI earlier, potentially preventing the vicious cycle of repeated hospitalizations, suffering, and chronic psychiatric morbidity.

## Appendix

Multimedia Appendix 1 Safety protocol for a research assistant.

Multimedia Appendix 2 Questions regarding adverse events and dropouts.

## Abbreviations

AIP: Adaptive Information Processing
ANOVA: analysis of variance
BSS: Beck Scale for Suicide Ideation
CSSRS: Columbia Suicide Severity Rating Scale
DES-II: Dissociative Experiences Scale-II
EMDR: Eye Movement Desensitization and Reprocessing
MDD: major depressive disorder
PTSD: posttraumatic stress disorder
RA: research assistant
RCT: randomized controlled trial
REDCap: Research Electronic Data Capture
SI: suicidal ideation
TAU: treatment as usual
TFT: trauma-focused therapy
