# Supplementary material for: Web-Based Eye Movement Desensitization and Reprocessing for Adults With Suicidal Ideation: Protocol for a Randomized Controlled Trial
Source: JMIR Res Protoc. 2021 Nov 4;10(11):e30711. doi: 10.2196/30711 (PMC8603176; doi:10.2196/30711)
Supplement: Multimedia Appendix 1 [file resprot_v10i11e30711_app1.pdf]

Safety Protocol for Research Assistant

Note: RA will refer to safety protocol at any point in workflow in case of patient emergency. This protocol can be entered at any of the points 1, 2, or 3. If any of points 1, 2, or 3 are entered, activate Emergency 0 (see next page).

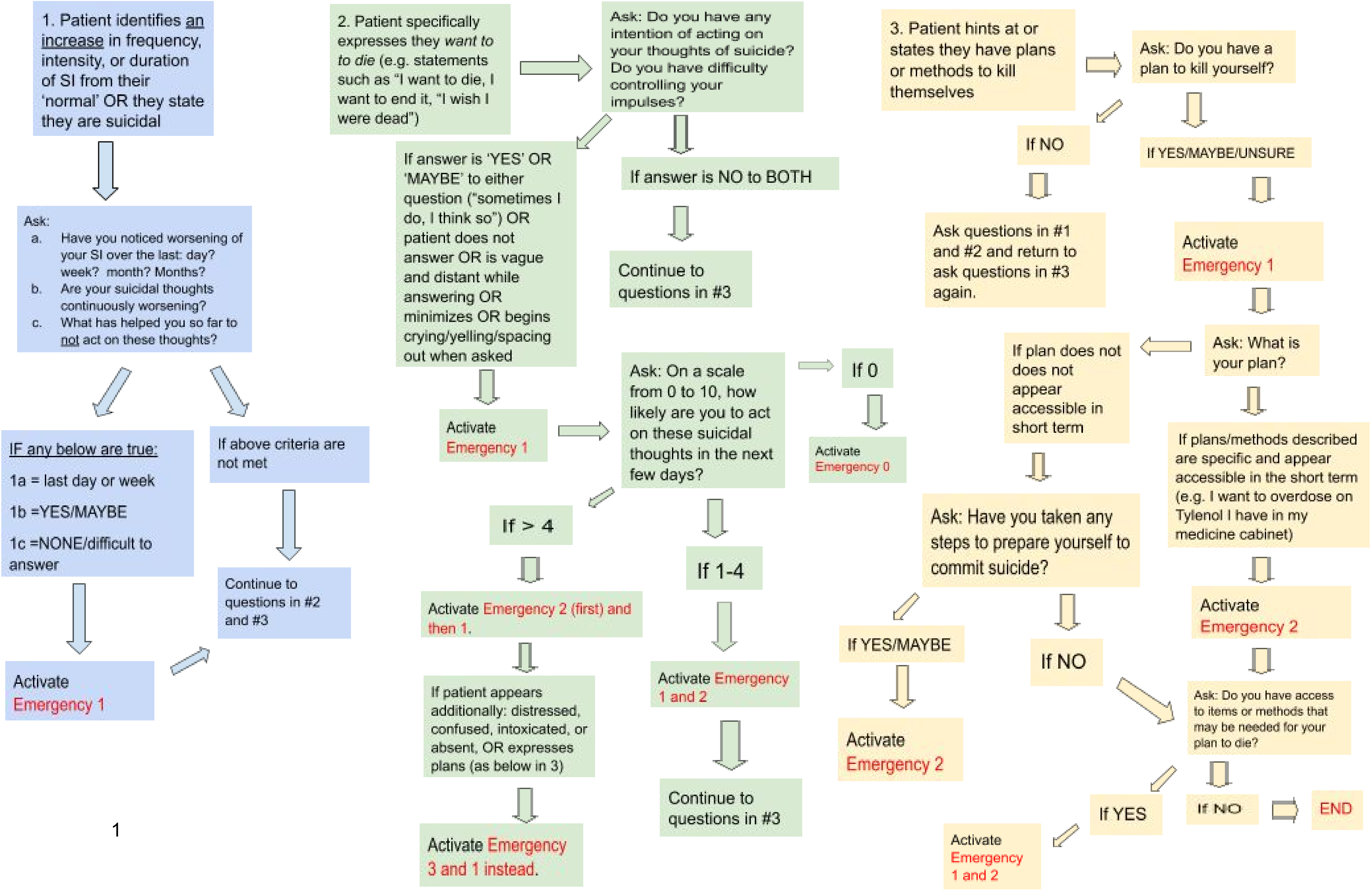

Safety Protocol for Research Assistant

Emergency 0:

If the participant is already enrolled in the study: Contact Drs. Burback or Winkler to discuss the case and arrange for non-urgent assessment time.

If patient is **not yet enrolled** in the study, encourage them to contact their psychiatrist/MHT for assessment.

For all: Ask the patient if they have an emergency/crisis plan and ask them to activate the plan.

Document the encounter, send copy of the chart note to treatment team (and if enrolled, study therapist). Debrief with study psychiatrists as soon as feasible.

Emergency 1: Contact Drs. Burback or Winkler to join the Zoom session urgently. Send both a text to their cell phones with the word "emergency." Email Drs to their AHS email with subject "emergency" and include ZOOM meeting ID and password of the patient in the email body.

Emergency 2:

"I am worried about your safety. **Can you go to the emergency room for an assessment?**

If NO/uncertain/wavering: activate Emergency 3 and 1

If YES, you may conclude the call, and activate Emergency 0

Emergency 3:

STEP1:"**Can you please confirm your name, address, and phone number with me?** (confirm if possible. If patient not cooperating, move to STEP2 below)

STEP 2:"**I am worried about your safety. Please stay online with me and keep your camera and microphone on as I call 911 to help you**"

Step 3: Call 911 and activate Emergency 1 as you keep the patient on the line.

If a patient hangs up suddenly, activate Emergency 1 and call 911 and provide the following information:

-state you are concerned about patient acting on SI and that they hung up on you as you were assessing them for it

-describe change in SI that patient provided

-describe plans if any

-describe intent if any

-provide patient's address,phone, emergency contact

IF patient does not want to wait and asks to hang up, return to Emergency 2 questions.

If patient asks to talk with them while they wait, specify that it must be unrelated to present SI or stressful situation. You may then chose to talk about

- weather

- last vacation they took

- a happy occurrence

-someone they are friends/family of with with NOT A COMPLICATED relationship

END: "**Thank you for answering my questions**" and:

If activating Emergency 1: **I will ask one of our doctors to join us right away to speak with us. Please wait with me as I do that.**

If activating Emergency 2: **I will let one of our therapists know about your difficulties and we'll arrange for a follow up appointment either with them or your usual treatment team. I will call you with the date/time of this appointment as soon as I know. (you may then end)**
