# Supplementary material for: Web-Based Eye Movement Desensitization and Reprocessing for Adults With Suicidal Ideation: Protocol for a Randomized Controlled Trial
Source: JMIR Res Protoc. 2021 Nov 4;10(11):e30711. doi: 10.2196/30711 (PMC8603176; doi:10.2196/30711)
Supplement: Multimedia Appendix 2 [file resprot_v10i11e30711_app2.pdf]

Questions related to Adverse Events and Dropouts for Virtual Eye Movement Desensitization and Reprocessing (EMDR) for Adults with Suicidal Ideation

Questions related to Adverse events (queried at the beginning of each session):

1) Describe the Adverse Event (AE)
   ____
   ____

2) Start date of AE ___________________

3) End date of AE ____________________

4) Severity
   - Mild
   - Moderate
   - Severe
5) Relationship to study treatment
   - Definitely related
   - Possibly related
   - Not related
   - Unknown
6) Action taken regarding study intervention
   - None
   - Discontinued permanently by therapist
   - Discontinued temporarily by therapist
   - Patient discontinued therapy
   - Protocol modified
   - Frequency of sessions changed
   - Hospital admission
   - Crisis management/safety planning
7) Outcome of AE
   - Resolved, no sequel
   - AE still present – no treatment
   - AE still present – being treated
   - Residual effects present – not treated
   - Residual effects present – treated
   - Death
   - Unknown
8) Expected
   - Yes

- No

9) Serious Adverse Event?
   - Yes
   - No
   - Unknown
10) Date of final study visit ___________________
11) Date of last known study intervention ____________________
12) Primary reason for terminating participation in the study
   - Completed study
   - Participant was determined after enrollment to be ineligible (Provide comments)
   - Participant withdrew consent
   - In the Investigator's opinion it was not in the participant's best interest to continue (Provide comments)
   - Adverse event (If checked, complete the AE form)
   - Death
   - Lost to follow-up
   - Other (Provide comments)
   - Unknown

<u>Questions related to dropouts:</u>

13) Did the participant revoke consent or is not able to proceed with the study for any reason?
   - Yes
   - No

14) What day did they stop participating in the study? _________________

15) Why did they stop participating?

    ____
    ____

16) Additional notes (did the client agree to complete any final measures, can we still use their previous data, was any follow-up/additional information requested, etc.)

    ____
    ____
    ____
    ____
